# Participant Experiences and Views of Odor and PrePex Device Removal Pain in a VMMC Pilot Study in Botswana

**DOI:** 10.1097/QAI.0000000000000765

**Published:** 2016-05-24

**Authors:** Adrian M. Musiige, Tigistu A. Ashengo, Galina Stolarsky, Rosinah T. Dialwa, Robert Manda, Conrad O. Ntsuape, Jerome Mafeni, Lesego Busang, Kelly Curran, Kenanao Motlhoiwa, Frank J. Mwangemi, Mainza Lukobo-Durrell, Mary T. Glenshaw

**Affiliations:** * Jhpiego, Gaborone, Botswana;; † Office of the Medical Director, Jhpiego, Washington, DC;; ‡ Division of HIV/AIDS and Tuberculosis, Center for Global Health, Centers for Disease Control and Prevention (CDC), Gaborone, Botswana;; § Department of HIV/AIDS Prevention and Care, Ministry of Health, Gaborone, Botswana;; ‖ African Comprehensive HIV/AIDS Partnerships (ACHAP), Gaborone, Botswana;; ¶ Monitoring and Evaluation Unit, Jhpiego, Washington, DC; and; # HIV and Infectious Diseases Unit, Jhpiego, Washington, DC.

**Keywords:** PrePex, odor, smell, pain, VMMC, male circumcision

## Abstract

**Objective:**

To assess participant experiences and perceptions of removal pain and odor associated with the PrePex device procedure.

**Methods:**

We analyzed data from a PrePex device pilot implementation study of 802 male participants aged 18–49 years at 2 clinics in Botswana, 2013. Study staff administered survey questions on device-related odor and assessed pain using visual analog scale scores categorized as no pain (0), mild (1–4), moderate (5–7), or severe pain (8–10).

**Results:**

Mean participant age was 27.7 (range = 18–48) years. Of the 802 participants, 751 (94%) reported to have noticed an unusual or unpleasant odor while wearing the device. Of these, 193 (26%) participants tried something to combat the odor. A total of 84 (10%) participants reported no pain, 655 (82%) mild pain, 48 (6%) moderate pain, and 15 (2%) severe pain at 2 minutes after device removal. Pain reports at 15 minutes after removal were 553 (69%) no pain, 247 (31%) mild pain, and 2 (0.25%) moderate pain, with no report of severe pain at this time point. Of 740 participants interviewed on day 42 after device placement, 678 (92%) were satisfied with the procedure and 681 (92%) would recommend it to another man considering circumcision, including 488 (66%) who would recommend it strongly.

**Conclusions:**

An unusual or unpleasant odor while wearing the PrePex device and mild self-limiting pain at device removal were common, but overall, these did neither seem to impair satisfaction nor deter participants from recommending PrePex to others, which could suggest good prospects for uptake of the device in this setting.

## BACKGROUND

The HIV prevention role of male circumcision has been demonstrated in several observational studies^1,2^ and 3 landmark clinical trials.^3–5^ The World Health Organization and Joint United Nations Programme on HIV/AIDS endorsed Voluntary Medical Male Circumcision (VMMC) for HIV prevention in 2007.^6^ Increasing data from longer posttrial follow-up studies show continued HIV protection.^7–9^

Botswana faces an estimated 18.5% HIV prevalence in the general population, with male circumcision prevalence estimated at 24% in the 10- to 64-year age group.^10^ Against this background, the Botswana Ministry of Health adopted VMMC for HIV prevention in 2009, targeting 80% of 13- to 49-year-old men, equivalent to 385,000 VMMC procedures by 2016. To increase demand and ease supply of VMMC, the Botswana Ministry of Health considered introduction of PrePex device and assessed it for safety and acceptability in 2013.

The PrePex device is one of a few adult male circumcision devices currently being studied in Africa. The device uses radial compression to cause ischemic necrosis of the foreskin. It obviates need to incise live tissue, to achieve hemostasis, and to insert skin sutures for wound closure. A key difference from other current adult devices is that necrotizing foreskin tissue distal to the PrePex device components remains in situ (attached) until device removal 5–7 days after placement. The procedure, as previously described elsewhere,^11^ is short and easy to learn by both nurses and physicians. Placement and removal require 3–5 minutes each. In addition, this procedure requires neither injectable anesthesia nor a sterile field.

Initial safety, efficacy, and acceptability profiles of the device were reported in Rwanda.^12^ In 2013, the device received World Health Organization prequalification for use in other country programs. Additional safety and acceptability data on the device have been reported from Uganda ^11,13^ and Kenya.^14^

The PrePex device has the potential to address 2 key barriers to the rollout and uptake of VMMC programs in sub-Saharan Africa: complexity of conventional surgical services and fear of pain. Although literature is yet to show cost benefits,^15–17^ the PrePex device could address some VMMC supply challenges by facilitating task shifting to nonphysicians^18^ to increase program reach. Fear of pain has been reported consistently across acceptability studies as a barrier for adolescents and adult men to seek VMMC services.^19–24^ A recent study from Kenya highlights lack of effective pain assessment in VMMC programs.^25^ PrePex eliminates pain or discomfort from injection of local anesthesia. One new barrier that may be introduced with the PrePex device is concern about odor while the device is in situ and also pain from device placement or removal. In 2 earlier PrePex studies in Uganda, 63%^11^ and 72%^13^ of participants reported to have noticed malodor. In one of these studies, 1% (3/300) thought that another person had noticed the odor. In addition, some experience a characteristic brief pain during device removal.^11,12^ If removal pain and odor do substantially affect user satisfaction, they could impair uptake of PrePex in some settings. There are limited detailed descriptive data on the effect of odor and PrePex device removal pain on client satisfaction with the PrePex procedure in the present literature. This report presents such data out of a PrePex safety and acceptability implementation pilot study in Botswana.

## METHODS

### Study Design

This was an open-label single-arm prospective study at 2 public clinics, approximately 10 km apart: Block 8 Clinic in Gaborone and Nkoyaphiri Clinic in a peri-urban location bordering Gaborone. Enrollment and data collection took place from May through September 2013. PrePex master trainers from Rwanda trained and certified providers (11 nurses and 4 physicians) who performed study procedures. On the device removal visit, providers assessed and documented participants' pain at 2 and 15 minutes after device removal using visual analog scale (VAS) scores, as described elsewhere.^11,26^ Trained staff interviewed participants on pain and odor experiences among other outcomes.

### Study Population and Eligibility

**FIGURE 1.**
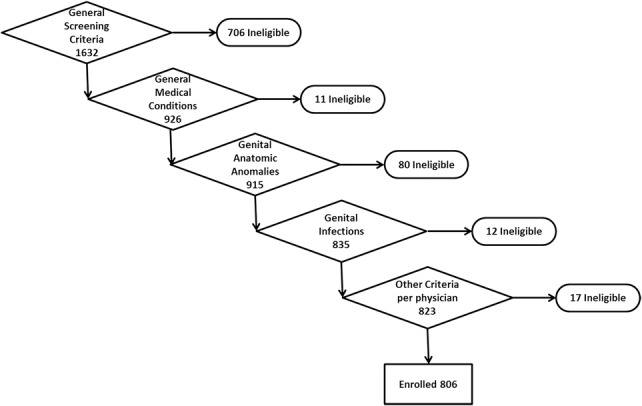
Participant screening outcomes.

Men from Gaborone and areas within 50 km around Gaborone who sought VMMC at the 2 clinics were invited to participate in the study. Inclusion criteria included age 18–49 years, uncircumcised, seeking VMMC at one of the study clinics, HIV negative, able to communicate in English or Setswana, able to understand study procedures, willingness to return for all scheduled follow-up visits at the study clinic, and willingness to commit some time to answer survey questions at each visit. Exclusion criteria included medical conditions, such as uncontrolled diabetes or hypertension, bleeding disorders, clinical anemia, and cognitive impairment. In addition, anatomical variations or genital anomalies, such as phimosis, praphimosis, or narrow prepuce, tight frenulum, urethral anomalies, such as hypospasdias and epispadias, hydrocele, scrotal hernia, or any genital anomaly were also excluded. Men with current genitourinary disease, ulcers from trauma or infection, genital warts, urethral discharge, balanitis, or posthitis were also excluded. Figure 1 shows a breakdown of screening outcomes.

### Study Enrollment Procedures

During the study period, VMMC clients at the study clinics were given information about the study at the reception and during group education and underwent study screening. Clients aged 18 years and older received further counseling and education about the PrePex device procedure. Eligible clients were offered choice between enrollment into the study for their circumcision or circumcision by routine conventional surgery. Those who opted for the PrePex device method and consented to participate were enrolled. Participants were withdrawn from further participation if none of the study device sizes (A–E) fitted them.

### Clinical and Follow-Up Procedures

The PrePex device procedures were performed per manufacturer's instructions,^27^ as described in detail in earlier literature.^11^ Nurses performed 83% of the placements and 82% of the removals; physicians performed the rest. Scheduled study visits were day 0 (enrollment and device placement), days 5–7 (device removal), day 14 (clinical review), and day 42 after placement to assess wound healing. Participants received transportation refund of approximately $7 USD on each scheduled visit. The study team made follow-up calls on days 0, 2, 28, and 35 or if a participant did not appear for a scheduled visit. Participants were provided with structured postoperative care instructions, including information on hygiene and wound care, review dates, and a 24-hour emergency contact number to reach a clinician any time after device placement as needed.

### Data Collection and Variables

Data were gathered with structured surveys on day 0 (immediately after device placement), day 7 (immediately after device removal), and day 42. Certified clinical providers assessed pain using a 10-point VAS 2 and 15 minutes after device removal. In addition, 6 focus group discussions (FGD) totaling 43 participants conducted upon study completion (day 42) elicited qualitative data regarding participants' perceptions of pain, odor, and satisfaction with the PrePex device method of circumcision.

### Statistical Considerations and Outcome Measures

A sample of 806 men for this pilot study was powered to detect the occurrence of adverse events, which was estimated to not exceed 2% from previous studies.^12,28^ As this study was the first to investigate rate of PrePex-related adverse events in the Botswana context, the sample size estimation was based on a confidence interval of 95% and a margin of error of ±1.5%. To account for between-site variation and within-site clustering, a design effect of 2 was used in the absence of empirical data. In addition, inflation of 20% in the sample size was used to account for men who withdrew after enrollment. This requisite sample size was also considered sufficient to make inferences on quantitative parameters for assessment of pain and odor outcome measures. Odor questions assessed any experience of unusual or unpleasant odor, odor description, timing (study day) that odor was first noticed, the level of discomfort of the participant from odor, perceived noticeability of the odor by other people, effect on activities of daily living, effect on ability to retain the PrePex device when in situ, and remedies to combat odor. We used Pearson χ^2^ test to evaluate the association between after placement hygiene practices and reporting of an unusual or unpleasant odor.

We categorized removal pain by scores as no pain (0), mild (1–4), moderate (5–7), or severe pain (8–10) at 2 and 15 minutes after device removal. We assessed proportions of participants reporting different levels of satisfaction with the PrePex device procedure and willingness to recommend it to a friend or family on 5-point scales. We used SPSS version 22.0 for descriptive and parametric tests of VAS pain scores and participant satisfaction and content analysis to assess perceptions about pain and odor from FGDs.

### Ethical Considerations

The study was approved by the Botswana Health Research Development Committee and the Institutional Review Boards of Johns Hopkins University School of Public Health and the US Centers for Disease Control and Prevention. Written informed consent was obtained from all participants.

## RESULTS

### Participant Profile

Teams at the 2 study clinics screened 1632 VMMC clients, of whom 806 (49%) were eligible and had the device placed. Of the 806, 535 (66%) were enrolled at Block 8 Clinic. Mean participant age was 27.7 (range = 18–48) years. Of the 826 screening failures, 706 (86%) failed general eligibility criteria (age 18–49 years, HIV negative, residence within 50 km radius around Gaborone, reliably reachable by phone, willing to participate). The other 120 (15%) were ineligible because of medical conditions other than HIV, distributed as follows: 11 (1%) uncontrolled diabetes or hypertension, 80 (10%) genital anatomical conditions, mainly phimosis or tight frenulum, 12 (1.5%) genital infections, and 17 (2%) for other prevailing conditions based on physician assessment.

### Follow-Up Rates

Nearly all participants (802/806, 99.5%) returned for device removal on schedule. Three participants removed part or the entire device by themselves before the scheduled removal date because of pain or discomfort and had conventional surgery to complete the removal of the foreskin. One participant excised his foreskin distal to the device with scissors at home on day 3 after placement because of odor leaving the device in place. Upon exclusion of these 4 participants from the analysis, the main analysis data set included 802 participants. Follow-up rates on days 14 and 42 after device placement were 785/802 (98%) and 740/802 (92%), respectively.

### Pain

Among the 802 participants assessed for pain at 2 minutes after device removal (day 7), 84 (10%) reported no pain, 655 (82%) reported mild pain, 48 (6%) reported moderate pain, and 15 (2%) reported severe pain. At 15 minutes after device removal, 553 (69%) reported no pain, 247 (31%) reported mild pain, 2 reported moderate pain, and none reported severe pain. After removal, 95% of all participants were satisfied with the method, including 61 (97%) of the 63 who reported pain scores 5 or higher 2 minutes after removal. At the day 42 interview, 678 (92%) participants reported satisfaction with the procedure, including 358 (48%) who reported being very satisfied (Table 1). Most of the participants reported either no difference or less than expected pain experience at removal. We found a negative correlation between pain score and satisfaction with the procedure (τ = −0.83). In a bivariate logistic regression, compared with those who experienced much less pain than expected, participants who reported more pain (odds ratio = 0.21, *P* < 0.001) or a lot more pain (odds ratio = 0.24, *P* < 0.001) than expected were significantly less likely to recommend PrePex.

**TABLE 1.**
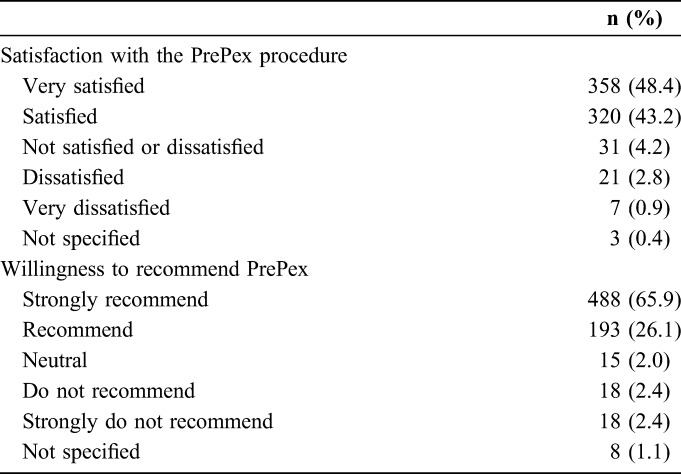
Distribution of Participant Ratings (n, %) of Satisfaction in Using the PrePex Device and Their Willingness to Recommend It to a Friend or Family, N = 740

### Odor

On the day 7 survey, 751/802 (94%) participants reported to have noticed an unusual or unpleasant odor while wearing the device. Most of them first noticed the odor on days 2, 3, or 4, 241 (33%), 292 (39%), and 146 (20%), respectively. Of the odor reporters, 343 (46%) participants were not disturbed by the odor, 299 (40%) reported “some discomfort,” while 109 (14%) felt “very uncomfortable” from the odor. Nineteen (2.5%) of the odor reporters thought another person, mainly a partner or another family member, also noticed the odor. We saw no significant association between reporting of an unusual or unpleasant odor and different reported penile washing practices (*P* = 0.78) while wearing the device (no washing, washing and air drying, washing and drying penis with a towel, or other).

Regarding activities of daily living among odor reporters, 539 (72%) reported no disruptions, 144 (19%) reported some disruption, and 41 (5%) reported “a lot” of disruption. Seventy (9%) participants reported having forfeited at least one activity because of the odor. Of these, 13 (19%) skipped school or work, 29 (41%) avoided to participate in community or social activity, 9 (13%) did not sleep or share usual space with partner, and 30 (43%) did not spend time or share space with family or friends because of odor. Of the participants who noticed an odor, 193 (26%) tried to combat it, commonly by frequent bathing or body perfumes. For 28 (15%) participants, the efforts did not help, whereas 111 (58%) and 52 (27%) thought the efforts helped “a little bit” and a lot to reduce the odor, respectively. Of all odor reporters, 95% would recommend PrePex. Of 7 (0.9%) men who were very dissatisfied with PrePex circumcision, 4 would strongly not recommend it. Overall, 93% of participants interviewed at the day 42 visit would recommend the procedure to another man, including 65.9% who would strongly recommend it (Table 1).

### Participant Perspectives on Odor and Pain From FGDs

In the FGDs, pain was described as considerable at device removal and lesser in the days after removal. One participant noted, “What I like about PrePex is that there is less pain.” However, alternative views were also raised, as some participants noted that more could be done to further address pain, citing earlier device removal (before days 5–7), stronger pre-procedure pain prophylaxis beyond the paracetamol or ibuprofen provided at device removal, and medically authorized absence (sick leave) after removal.

The extreme of unfavorable descriptions of the odor is embodied in one participant's view that “it smelled like rotten eggs.” Participants also raised the need for better ways to curb the odor, as expressed by another that “… they should try something that reduces the smell so that when you are around people they will not be able to smell it. As for pain there are some pills; if you feel pain then you can take some but there is nothing for this smell. It's a bit of a problem.”

## DISCUSSION

This study looked at the 2 clinical aspects of PrePex circumcision: odor and pain. A higher proportion of participants reported having noticed an “unusual or unpleasant smell” and a lower proportion of patients believed someone else noticed they had an odor, compared with previous studies from Uganda.^11,13^ From participant FGDs at the conclusion of the clinical review period, there was general agreement that there was noticeable odor when wearing the PrePex device. Except for the one participant who explained his self-removal of the foreskin tissue as an effort to combat odor, the study team did not observe need or pressing participant requests to remove the device and foreskin tissue ahead of schedule because of odor. However, the finding that 1 in 4 participants who reported odor tried one or more measures to combat it indicates a strong desire for odor to be mitigated. Aptly, a recent (March 2014) device manufacturer clinical update recommends daily cleansing between the foreskin and glans with 1% chlorhexidine solution to mitigate the odor. Systematic evaluation of this intervention in different settings might be useful to further validate its efficacy. Given that we saw no obvious association between hygiene practices and likelihood to report or not report odor, further investigation into the characteristics of odor reporters and non-odor reporters is still relevant.

The finding that participants who reported more pain than they expected were less likely to recommend the procedure calls for careful preoperative messaging to ensure that clients receive accurate information about pain expectations. Thus, we recommend that these messages do articulate a likelihood of some degree of pain, particularly at the time of device removal, but which is brief and self-limiting at 2–15 minutes in almost all cases. Qualitative results of this study regarding expansion of pain management options should be duly considered in program implementation.

High participant satisfaction ratings observed in this study despite the reported removal pain and odor experiences suggest that although these symptoms are of concern, they do not seem likely to overshadow favorable attributes of the PrePex procedure from the perspective of a typical client in this setting.

One limitation of our study may be the fact that most participants were from an urban setting, and their opinions and perceptions about pain and odor experiences could differ from those from a typical rural population. In addition, the wording of our odor outcome questions in our survey (phrased as unusual or unpleasant odor) might explain the relatively higher proportion of odor reporters. Although some FGD participants reported the odor to be offensive, we are unable to quantify participants who noticed unusual versus unpleasant odor.

Findings of this study collaborate with those from previous studies to affirm findings that odor while wearing the PrePex device is common, infrequently noticeable to another person, and does neither necessitate early device removal nor deter users from recommending the method to others as per this study. Odor and removal pain may not be major barriers to uptake of PrePex in this setting, though additional work to optimize odor prevention and pain management protocols would be welcome. Accurate messaging on odor (PrePex) and pain (PrePex and conventional surgery) should be part of client counseling before VMMC to ensure that clients have appropriate expectations ahead of the procedure.

## References

[1] O'Farrell N Egger M Circumcision in men and the prevention of HIV infection: a “meta-analysis” revisited. Int J STD AIDS. 2000;11:137–142. 10726934 10.1258/0956462001915480

[2] Weiss HA Quigley MA Hayes RJ Male circumcision and risk of HIV infection in sub-Saharan Africa: a systematic review and meta-analysis. AIDS. 2000;14:2361–2370. 11089625 10.1097/00002030-200010200-00018

[3] Auvert B Taljaard D Lagarde E Randomized, controlled intervention trial of male circumcision for reduction of HIV infection risk: the ANRS 1265 Trial. PLoS Med. 2005;2:e298. 16231970 10.1371/journal.pmed.0020298 PMC1262556

[4] Bailey RC Moses S Parker CB Male circumcision for HIV prevention in young men in Kisumu, Kenya: a randomised controlled trial. Lancet. 2007;369:643–656. 17321310 10.1016/S0140-6736(07)60312-2

[5] Gray RH Kigozi G Serwadda D Male circumcision for HIV prevention in men in Rakai, Uganda: a randomised trial. Lancet. 2007;369:657–666. 17321311 10.1016/S0140-6736(07)60313-4

[6] WHO, UNAIDS. New Data on Male Circumcision and HIV Prevention: Policy and Programme Implications. Montreux, Switzerland: WHO/UNAIDS; 2007:6–8.

[7] Auvert B Taljaard D Rech D Association of the ANRS-12126 male circumcision project with HIV levels among men in a South African township: evaluation of effectiveness using cross-sectional surveys. PLoS Med. 2013;10:e1001509. 24019763 10.1371/journal.pmed.1001509 PMC3760784

[8] Gray R Kigozi G Kong X The effectiveness of male circumcision for HIV prevention and effects on risk behaviors in a posttrial follow-up study. AIDS. 2012;26:609–615. 22210632 10.1097/QAD.0b013e3283504a3f PMC4296667

[9] Mehta SD Moses S Agot K The long-term efficacy of medical male circumcision against HIV acquisition. AIDS. 2013;27:2899–2907. 23835501 10.1097/01.aids.0000432444.30308.2d

[10] Statistics Botswana (2014). Botswana Aids Impact Survey (BAIS IV) 2013. Available at: http://1govportal.imexsystems.net/en-gb/Documents/Ministry%20of%20State%20President/NACA/Botswana%20AIDS%20Impact%20Survey%20IV%20Report.pdf. Accessed August 25, 2015.

[11] Galukande M Duffy K Bitega JP Adverse events profile of PrePex a non-surgical device for adult male circumcision in a Ugandan urban setting. PLoS One. 2014;9:e86631. 24489754 10.1371/journal.pone.0086631 PMC3904949

[12] Bitega JP Ngeruka ML Hategekimana T Safety and efficacy of the PrePex device for rapid scale-up of male circumcision for HIV prevention in resource-limited settings. J Acquir Immune Defic Syndr. 2011;58:e127–e134. 21909032 10.1097/QAI.0b013e3182354e65

[13] Kigozi G Musoke R Watya S The safety and acceptance of the PrePex device for non-surgical adult male circumcision in Rakai, Uganda. A non-randomized observational study. PLoS One. 2014;9:e100008. 25144194 10.1371/journal.pone.0100008 PMC4140666

[14] Feldblum PJ Odoyo-June E Obiero W Safety, effectiveness and acceptability of the PrePex device for adult male circumcision in Kenya. PLoS One. 2014;9:e95357. 24788898 10.1371/journal.pone.0095357 PMC4006910

[15] Duffy K Galukande M Wooding N Reach and cost-effectiveness of the PrePex device for safe male circumcision in Uganda. PLoS One. 2013;8:e63134. 23717402 10.1371/journal.pone.0063134 PMC3661578

[16] Njeuhmeli E Kripke K Hatzold K Cost analysis of integrating the PrePex medical device into a voluntary medical male circumcision program in Zimbabwe. PLoS One. 2014;9:e82533. 24801515 10.1371/journal.pone.0082533 PMC4011574

[17] Obiero W Young MR Bailey RC The PrePex device is unlikely to achieve cost-savings compared to the forceps-guided method in male circumcision programs in sub-Saharan Africa. PLoS One. 2013;8:e53380. 23349708 10.1371/journal.pone.0053380 PMC3549910

[18] Mutabazi V Kaplan SA Rwamasirabo E One-arm, open-label, prospective, cohort field study to assess the safety and efficacy of the PrePex device for scale-up of nonsurgical circumcision when performed by nurses in resource-limited settings for HIV prevention. J Acquir Immune Defic Syndr. 2013;63:315–322. 23466648 10.1097/QAI.0b013e31828e6412

[19] Evens E Lanham M Hart C Identifying and addressing barriers to uptake of voluntary medical male circumcision in Nyanza, Kenya among men 18-35: a qualitative study. PLoS One. 2014;9:e98221. 24901226 10.1371/journal.pone.0098221 PMC4047024

[20] Lukobo MD Bailey RC Acceptability of male circumcision for prevention of HIV infection in Zambia. AIDS Care. 2007;19:471–477. 17453585 10.1080/09540120601163250

[21] Ngalande RC Levy J Kapondo CP Acceptability of male circumcision for prevention of HIV infection in Malawi. AIDS Behav. 2006;10:377–385. 16736112 10.1007/s10461-006-9076-8

[22] Plotkin M Castor D Mziray H “Man, what took you so long?” Social and individual factors affecting adult attendance at voluntary medical male circumcision services in Tanzania. Glob Health Sci Pract. 2013;1:108–116. 25276521 10.9745/GHSP-D-12-00037 PMC4168557

[23] Bailey RC Muga R Poulussen R The acceptability of male circumcision to reduce HIV infections in Nyanza Province, Kenya. AIDS Care. 2002;14:27–40. 11798403 10.1080/09540120220097919

[24] Ssekubugu R Leontsini E Wawer MJ Contextual barriers and motivators to adult male medical circumcision in Rakai, Uganda. Qual Health Res. 2013;23:795–804. 23515302 10.1177/1049732313482189

[25] Reed J Grund J Liu Y Evaluation of loss-to-follow-up and post-operative adverse events in a voluntary medical male circumcision program in Nyanza Province, Kenya. J Acquir Immune Defic Syndr. 2015. 10.1097/QAI.0000000000000535 25942466

[26] Williamson A Hoggart B Pain: a review of three commonly used pain rating scales. J Clin Nurs. 2005;14:798–804. 16000093 10.1111/j.1365-2702.2005.01121.x

[27] PrePex (2014). PrePex User Manual. Available at: http://prepex.com/wp-content/uploads/2014/05/User-Manual-A4-Rev14.pdf. Accessed August 25, 2015.

[28] Mutabazi V Kaplan SA Rwamasirabo E HIV prevention: male circumcision comparison between a nonsurgical device to a surgical technique in resource-limited settings: a prospective, randomized, nonmasked trial. J Acquir Immune Defic Syndr. 2012;61:49–55. 22739133 10.1097/QAI.0b013e3182631d69

